# Time-dependent diffusion-weighted imaging assessment of tumor grading and *isocitrate dehydrogenase* genotypes in adult-type diffuse gliomas

**DOI:** 10.1007/s11604-025-01936-w

**Published:** 2026-01-05

**Authors:** Kiyohisa Kamimura, Tsubasa Nakano, Masanori Nakajo, Junki Kamizono, Tomohito Hasegawa, Daiki Tobo, Akie Mukai, Yoshiki Kamimura, Fumitaka Ejima, Hiroaki Nagano, Koji Takumi, Masatoyo Nakajo, Nayuta Higa, Hajime Yonezawa, Ryosuke Hanaya, Mari Kirishima, Akihide Tanimoto, Hirokazu Otsuka, Daisuke Hirahara, Hiroshi Imai, Thorsten Feiweier, Takashi Yoshiura

**Affiliations:** 1 Department of Advanced Radiological Imaging, Kagoshima University Graduate School of Medical and Dental Sciences, 8-35-1, Sakuragaoka, Kagoshima 890-8544 Japan; 2 Department of Radiology, Kagoshima University Graduate School of Medical and DentalSciences, 8-35-1, Sakuragaoka, Kagoshima 890-8544 Japan; 3 Departmentof Neurosurgery, Kagoshima University Graduate School of Medicaland Dental Sciences, 8-35-1, Sakuragaoka, Kagoshima 890-8544 Japan; 4 Department of Pathology, KagoshimaUniversity Graduate School of Medical and Dental Sciences, 8-35-1, Sakuragaoka, Kagoshima 890-8544 Japan; 5 Department of Radiological Technology, Kagoshima University Hospital, 8-35-1, Sakuragaoka, Kagoshima 890-8544 Japan; 6 Department of AI Research Lab, Harada Academy, 2-54-4, Higashi-Tanayama, Kagoshima 891-0113 Japan; 7 Siemens Healthcare K.K., Gate City Osaki West Tower, 1-11-1, Shinagawa-ku, Tokyo, Osaki 141-8644 Japan; 8 Research & Clinical Translation, Magnetic Resonance, Siemens Healthineers AG, Allee am Roethelheimpark 2, Erlangen, 91052 Germany

**Keywords:** Adult-type diffuse glioma, *Isocitrate dehydrogenase* genotypes, MRI, Time-dependent diffusion, Tumor grading

## Abstract

**Background:**

This study aimed to investigate the usefulness of time-dependent diffusion magnetic resonance imaging (MRI) parameters compared with the conventional apparent diffusion coefficient (ADC) in distinguishing tumor grade and isocitrate dehydrogenase (IDH) genotypes of adult-type diffuse gliomas.

**Methods:**

This retrospective study included 102 patients with adult-type diffuse gliomas. ADC maps obtained using diffusion-weighted imaging at short (7.1 ms) and long (44.5 ms) diffusion times (ADC7.1ms and ADC44.5ms) and maps of ADC changes (cADC) and relative ADC changes (rcADC) between the two diffusion times were generated. The mean, 5th, and 95th percentile values of each parameter were compared between low-grade (LGGs) and high-grade gliomas (HGGs) and between IDH-mutant and IDH-wildtype gliomas. The discriminative performance was assessed using receiver operating characteristic (ROC) analysis, and correlation with Ki-67 labeling index (Ki-67LI) was assessed using Spearman’s rank correlation. Multivariable logistic regression analyses were conducted to predict HGGs and IDH-wildtype gliomas.

**Results:**

In HGGs, the mean and 5th percentile values of ADC44.5ms and ADC7.1ms were significantly lower, whereas cADC and rcADC indices were significantly higher than those in LGGs. Performance of the mean rcADC (area under the ROC curve: 0.925; 95% confidence interval: 0.855–0.967) was significantly better than any index of conventional ADCs for tumor grade classification. The mean rcADC demonstrated the strongest correlation with Ki-67LI (ρ = 0.542, p < 0.0001). Moreover, the 95th percentile of rcADC was an independent predictor of IDH-wildtype gliomas after adjustment for age and sex, was useful for distinguishing IDH-wildtype from IDH-mutant gliomas

**Conclusions:**

The mean rcADC showed the strongest correlation with the Ki-67 LI and achieved better diagnostic performance than conventional PGSE-based ADC for differentiating LGGs from HGGs. In multivariable analyses, the mean and 95th percentile of rcADC were identified as independent predictors of HGGs and IDH-wildtype gliomas, respectively.

**Supplementary Information:**

The online version contains supplementary material available at 10.1007/s11604-025-01936-w.

## Introduction

Adult-type diffuse gliomas account for a large proportion of primary brain tumors and remain challenging to diagnose and treat. Traditionally, these tumors have been classified as low-grade gliomas (LGGs) and high-grade gliomas (HGGs) according to their histological features, with LGGs generally associated with a more favorable prognosis [1]. The fifth edition of the World Health Organization (WHO) Classification of Tumors of the Central Nervous System emphasizes the importance of molecular and genetic profiles in the diagnosis of diffuse gliomas [2]. The mutation status of the *isocitrate dehydrogenase (IDH)* gene is a crucial factor in tumor development. The *IDH*-mutant gliomas demonstrate a less aggressive biological behavior and are more responsive to chemotherapy, which is related to a more favorable prognosis [3]. Moreover, patients whose tumors have chromosome arms 1p and 19q codeletion (*1p/19q* codeletion) a feature that is frequently observed in oligodendroglioma survive longer than those with tumors that lack this deletion [4]. A small number of *IDH*-mutant gliomas are classified as HGGs, making the differentiation between LGGs and HGGs equally important [2]. The Ki-67 labeling index (LI), a marker of tumor cell proliferation, is strongly associated with tumor differentiation, invasiveness, and patient prognosis [5, 6]. A previous study has revealed the Ki-67 LI as a crucial prognostic factor in astrocytomas [7]. Biopsy and histopathological analysis are the gold standard for glioma classification and molecular feature detection [2]. However, these procedures are invasive and may lead to sampling errors due to intratumoral heterogeneity [8]. Conventional MRI is a noninvasive technique; however, it only provides limited information for an accurate diagnosis [9−13].

Diffusion-weighted imaging (DWI) and apparent diffusion coefficient (ADC) measurements provide important information about microstructural organization. ADC values exhibit an inverse correlation with tumor cellularity [9]. Studies have demonstrated significantly higher ADC values obtained from DWI for patients with LGG than those with HGG due to lower cellularity and a reduced nuclear-to-cytoplasmic ratio thereby enabling the use of DWI for glioma grading [10−13]. Moreover, the ADC values are significantly higher for *IDH*-mutant glioma than for *IDH*-wildtype glioma [14−16]. Conversely, *1p/19q* codeletion status, the ADC value remains unidentified [14−16]. Diffusion time is a fundamental parameter in DWI and represents the observation time of diffusion. Spatial barriers, such as cell membranes, restrict the movement of water molecules (restricted diffusion) in biological tissues. Under these conditions, ADC values increase as the diffusion time decreases [17–20], since a shorter diffusion time reduces the likelihood of molecular collisions with these barriers. Conventional pulsed-gradient spin-echo (PGSE) DWI sequences require a long diffusion time to attain high b-values because of the limitation in maximum gradient strength [21, 22]. Moreover, the 180° pulse lasts several milliseconds; thus, the diffusion time in PGSE has a lower limit, even if the gradient strength is unrestricted. Thus, investigating the effect of diffusion time on ADCs using a clinical MRI scanner is challenging. The oscillating-gradient spin-echo (OGSE) DWI sequence is a novel diffusion encoding technique [17] that uses rapidly oscillating gradients instead of the prolonged diffusion-sensitizing gradients utilized in the PGSE method, thereby enabling shorter diffusion times. Studies have investigated the diffusion time dependence of the ADC by combining the PGSE and OGSE methods, which is an approach known as time-dependent diffusion MRI [23–27]. Time-dependent diffusion MRI can provide additional information about restricted diffusion within the tissue microstructure.

To the best of our knowledge, no previous study has evaluated how diffusion time affects the diagnostic performance of ADC for distinguishing *IDH*-mutant from *IDH*-wildtype adult-type diffuse gliomas. Only one study has reported the differentiation of low- and high-grade intra-axial brain tumors using time-dependent diffusion MRI [24]. The present study extends the scope by investigating the correlations between time-dependent diffusion MRI parameters and a broader range of tumor characteristics, including pathology, grade, *IDH* mutation status, *1p/19q* codeletion, and Ki-67 expression. Further, this study aims to assess the potential of time-dependent diffusion MRI in distinguishing not only LGGs from HGGs but also *IDH*-mutant from *IDH*-wildtype gliomas.

## Materials and methods

### Patients

Our institutional review board (approval no. 220126), which waived the requirement for written informed consent due to its retrospective design, approved this study. This study included consecutive cases of pathologically confirmed adult-type diffuse gliomas that were classified according to the 2021 CNS WHO classification [2] at our institution from January 2019 to December 2024. The exclusion criteria were (a) absence of preoperative MRI, including DWI with both OGSE and PGSE sequences; (b) inadequate image quality; or (c) history of surgical resection or irradiation. The largest tumor was subject to analysis for patients with multiple lesions.

## Pathological/molecular diagnosis

All *IDH*-mutant and *1p/19q-*codeleted, *IDH*-mutant, and *IDH*-wildtype adult-type diffuse gliomas were diagnosed employing an integrated approach that combined histological assessment with a glioma-tailored next-generation sequencing panel that was developed at our institution [28]. The Ki-67 LI was quantified with immunohistochemical staining and defined as the percentage of malignant cell nuclei demonstrating positive staining [7]. The glioblastomas, *IDH*-wildtype, were classified as CNS WHO grade 4; astrocytomas, *IDH*-mutant, were classified as CNS WHO grade 2, 3, or 4; and oligodendrogliomas, *IDH*-mutant and *1p/19q-*codeleted, were classified as CNS WHO grade 2 or 3.

## MRI acquisition

All patients underwent imaging using a 3T MR scanner (MAGNETOM Prisma; Siemens Healthineers AG, Forchheim, Germany) equipped with a 20-channel head-neck radiofrequency receive coil, featuring a maximum gradient amplitude of 80 mT/m and a maximum slew rate of 200 T/m/s for each gradient axis. Moreover, DWI was acquired using research sequences for OGSE DWI with b-values of 0 and 1,500 s/mm² (number of averages: 1 and 4, respectively) and three diffusion encoding directions. OGSE diffusion encoding was performed using trapezoid-sine waveforms [29]. An effective diffusion time (Δ_eff_) of 7.1 ms (frequency = 50 Hz; diffusion gradient pulse duration [δ] = 8.5 ms) was applied. Moreover, PGSE DWI was obtained with b-values of 0 and 1,500 s/mm² (number of averages: 1 and 4, respectively) and three diffusion encoding directions. The Δ_eff_ for the PGSE encoding was 44.5 ms (with a diffusion gradient separation [Δ] of 59.8 ms and δ of 46.1 ms). Both sequences were acquired utilizing the same parameters: repetition time (TR), 4,600 ms; echo time (TE), 120 ms; field of view (FOV), 230 × 230 mm^2^; matrix size, 72 × 72; and slice thickness, 5 mm. The acquisition times were 1 min 31 s for PGSE DWI and 1 min 39 s for OGSE DWI. Supplementary Fig. 1 illustrates the pulse sequence diagrams for both PGSE and OGSE.

Precontrast fluid-attenuated inversion recovery (FLAIR) images and postcontrast 2D T1-weighted spin-echo images were acquired (Supplementary Table 1). These images served as an anatomical reference for delineating regions of interest (ROIs). Our standard MRI protocol for central nervous system lesions included precontrast sequences as follows: 2D T1-weighted spin-echo imaging, 2D T2-weighted turbo spin-echo imaging, and 3D susceptibility-weighted imaging. The precontrast T1-weighted images were utilized to confirm contrast enhancement.

## Creating diffusion parametric maps

ADC values were measured following the assumption of mono-exponential signal decay between lower and higher b-values.

The ADC change (cADC) and relative ADC change (rcADC) between OGSE (short diffusion time) and PGSE (long diffusion time) were assessed based on previous studies [23, 24]. The pixel-by-pixel calculations were performed to develop cADC and rcADC maps using the following formulas:

$$\begin{aligned} \mathrm{cADC}&= \mathrm{ADC}_{7.1{\mathrm{ms}}} - \mathrm{ADC}_{44.5{\mathrm{ms}}} \\ \mathrm{rcADC}& = \frac{(\mathrm{ADC}_{7.1{\mathrm{ms}}} - \mathrm{ADC}_{44.5{\mathrm{ms}}})}{\mathrm{ADC}_{44.5{\mathrm{ms}}}} \times 100 (\%) \end{aligned}$$

Where ADC_7.1ms_ and ADC_44.5ms_ represent the ADC values acquired using the OGSE and PGSE sequences, respectively.

## ROI-based measurement

Commercially available software (Vitrea; Canon Medical Systems Corporation) was used for all image analyses. ADC maps were aligned with the postcontrast T1-weighted and FLAIR images using rigid body registration. Two independent radiologists (T.N. and J.K., with 10 and 8 years of radiological experience, respectively), who were blinded to the patients’ clinical and pathological information, conducted an ROI analysis. A solid tumor component with significant enhancement was delineated using transverse contrast-enhanced T1-weighted images, whereas a nonenhancing solid tumor component was delineated utilizing transverse FLAIR images. The ROIs excluded tumor necrosis, cystic components, surrounding edema, macroscopic hemorrhage, and calcification. Subsequently, these ROIs were transferred to the corresponding ADC, cADC, and rcADC maps, which had been automatically registered to the postcontrast T1-weighted image employing Vitrea.

The mean ADC_44.5ms_ (ADC_44.5 ms_^mean^), ADC_7.1 ms_ (ADC_7.1 ms_^mean^), cADC (cADC^mean^), and rcADC (rcADC^mean^) values were measured for the entire ROI. The minimum ADC value of gliomas correlated well with tumor cellularity, and that of HGGs was significantly lower than that of LGGs [30]. Therefore, we aimed to investigate the minimum and maximum ADC values of gliomas. In this study, ADC values were calculated pixel by pixel. To minimize the effects of noise, the 5th and 95th percentiles of each ADC distribution were identified, as this approach provided the lowest and highest robust values [30]. Therefore, the 5th and 95th percentile values of the ADC_44.5 ms_ (ADC_44.5 ms_^5th^ and ADC_44.5ms_^95th^), ADC_7.1ms_ (ADC_7.1ms_^5th^ and ADC_7.1ms_^95th^), cADC (cADC^5th^ and cADC^95th^), and rcADC (rcADC^5th^ and rcADC^95th^) were calculated.

### Statistical analysis

The D’Agostino–Pearson normality test was used to assess the normality assumption for all parameters in all groups. Among the three groups, the one-way analysis of variance (ANOVA) was used to compare differences in age, Ki-67 LI, and time interval between MRI and surgery, whereas the chi-square test was utilized to compare differences in sex and CNS WHO grade. The intraclass correlation coefficient (ICC) was used to assess the interobserver agreement on parametric measurements, with an ICC of > 0.74 indicating excellent agreement [31]. Measurements from the two observers were averaged for each case and applied for subsequent analysis. Tumors were grouped based on the histopathological results. The mean values, as well as the 5th and 95th percentile values of the ADC_44.5ms_, ADC_7.1ms_, cADC, and rcADC, were compared as follows. For parameters with nonnormal distribution, the diffusion parameters between two groups were compared using the Kruskal–Wallis test; whereas, the diffusion parameters among three groups were compared using the Kruskal–Wallis test with the Bonferroni correction. Further, for parameters with normal distribution, the comparison between two groups was performed using ANOVA; whereas, comparison among three groups was conducted using ANOVA with the Bonferroni correction. A receiver operating characteristic (ROC) curve analysis was conducted to identify the optimum threshold for tumor differentiation and to measure the area under the ROC curve (AUC), sensitivity, specificity, and accuracy for determining *IDH*-wildtype gliomas or HGGs. Optimal ADC_44.5ms_, ADC_7.1ms_, cADC, and rcADC indices were selected. DeLong’s test was used to compare the AUCs of the best-performing indices. The Bonferroni correction was applied for multiple comparisons. Further, we conducted multivariable logistic regression analyses to predict HGGs and *IDH*-wildtype gliomas. Candidate variables (age, sex, one conventional PGSE-based ADC index, and one rcADC index) were selected a priori based on clinical relevance and their diagnostic performance in the univariable analyses, and all selected variables were entered simultaneously (forced entry) in each multivariable model. As an internal validation, we assessed the stability of the model coefficients using 1,000 bootstrap resampling. In addition, the Hosmer–Lemeshow test was used to evaluate the calibration of the model. Spearman’s rank correlation analysis was conducted to assess the association between the diffusion parameters and Ki-67 LI. The correlation was considered little or no relationship if 0 ≤ ρ < 0.25, fair if 0.25 ≤ ρ < 0.5, moderate to good if 0.5 ≤ ρ < 0.75, and very good to excellent if 0.75 ≤ ρ [32]. Statistical analysis was conducted using a commercially available software package (MedCalc, version 15.10.0; MedCalc statistical software, IBM, version 28.0.1.0; IBM SPSS Statistics). Furthermore, statistical significance was set at p values of < 0.05.

## Results

### Patients

**Fig. 1:**
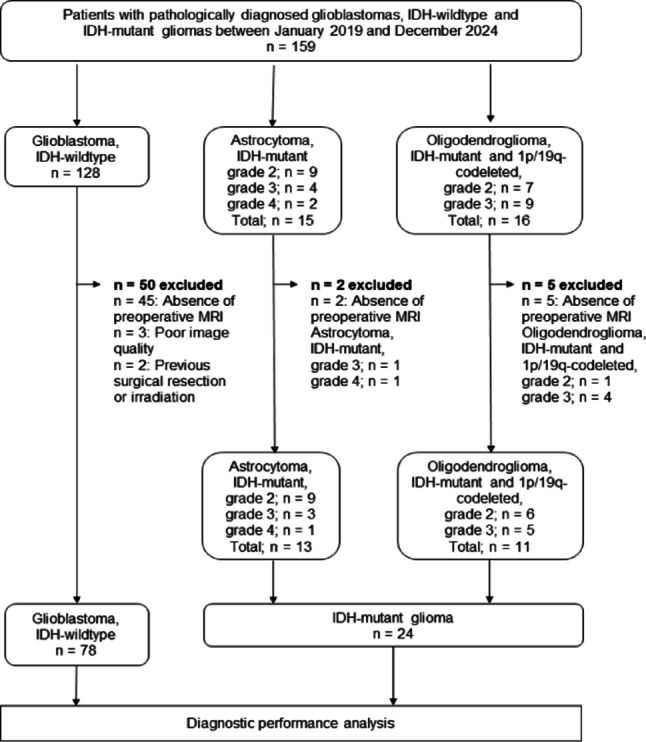
Diagram indicating the inclusion and exclusion criteria and the flow of the inclusion of eligible patients in this study

**Fig. 2:**
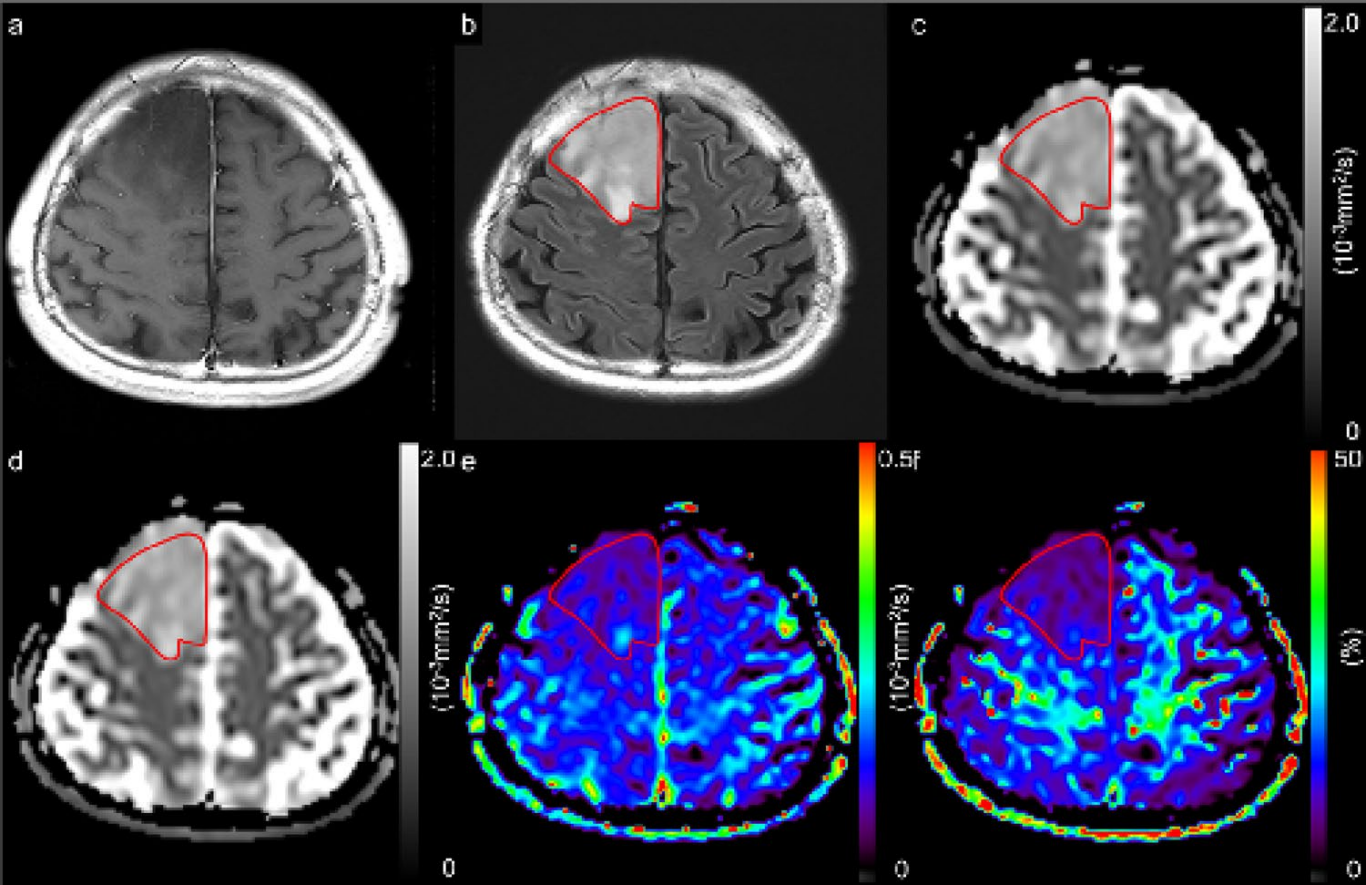
A 53-year-old male patient with astrocytoma, *isocitrate dehydrogenase*-mutant, CNS WHO grade 2. A contrast-enhanced T1-weighted image demonstrated no contrast enhancement (**a**). A fluid-attenuated inversion recovery image with a ROI (solid line) (**b**). ADC maps derived from PGSE DWI at a Δ_eff_ of 44.5 ms (**c**) and from OGSE DWI at a Δ_eff_ of 7.1 ms (**d**). Maps of cADC (e) and rcADC (f) between PGSE and OGSE. Low cADC and rcADC levels in the tumor were observed. The mean ADC_44.5ms_, ADC_7.1ms_, cADC, and rcADC values are 1.271 × 10^− 3^ mm^2^/s, 1.323 × 10^− 3^ mm^2^/s, 0.052 × 10^− 3^ mm^2^/s, and 4.05%, respectively *Δ*_*eff*_ effective diffusion time, *ADC* apparent diffusion coefficient, *cADC* apparent diffusion coefficient change, *DWI* diffusion-weighted imaging, *OGSE* oscillating-gradient spin-echo, *PGSE* pulsed-gradient spin-echo, *rcADC* relative apparent diffusion coefficient change

**Fig. 3:**
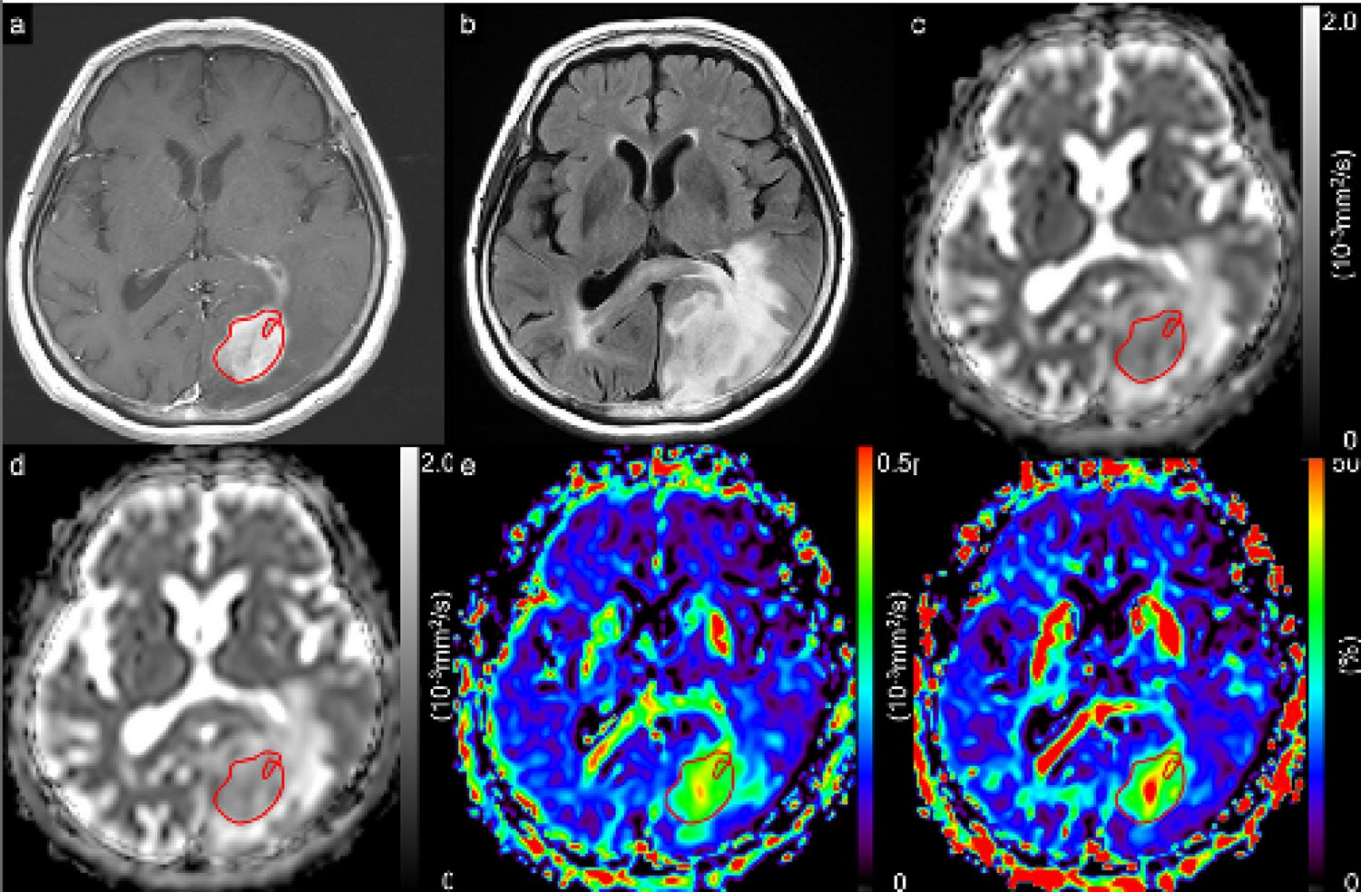
A 70-year-old female patient with glioblastoma, *isocitrate dehydrogenase*-wildtype, CNS WHO grade 4. A contrast-enhanced T1-weighted image with a ROI (solid line) (**a**). A fluid-attenuated inversion recovery image (**b**). ADC maps derived from PGSE DWI at a Δ_eff_ of 44.5 ms (**c**) and from OGSE DWI at a Δ_eff_ of 7.1 ms (**d**). Maps of cADC (**e**) and rcADC (**f**) between PGSE and OGSE. High cADC and rcADC levels in the tumor were observed. The mean ADC_44.5ms_, ADC_7.1ms_, cADC, and rcADC values are 0.988 × 10^− 3^ mm^2^/s, 1.118 × 10^− 3^ mm^2^/s, 0.130 × 10^− 3^ mm^2^/s, and 13.9%, respectively *Δ*_*eff*_ effective diffusion time, *ADC* apparent diffusion coefficient, *cADC* apparent diffusion coefficient change, *DWI* diffusion-weighted imaging, *OGSE* oscillating-gradient spin-echo, *PGSE* pulsed-gradient spin-echo, *rcADC* relative apparent diffusion coefficient change

In this study, 159 consecutive patients, comprising (128 with glioblastoma, *IDH*-wildtype, CNS WHO grade 4; 9 with astrocytoma, *IDH*-mutant, CNS WHO grade 2; 4 with astrocytoma, *IDH*-mutant, CNS WHO grade 3; 2 with astrocytoma, *IDH*-mutant, CNS WHO grade 4; 7 with oligodendroglioma, *IDH*-mutant and *1p/19q-*codeleted, CNS WHO grade 2; and 9 with oligodendroglioma, *IDH*-mutant and *1p/19q-*codeleted, CNS WHO grade 3) were considered for inclusion. This study excluded 57 patients because of the absence of preoperative MRI, including both OGSE and PGSE DWI scans, poor image quality caused by artifacts in DWIs, or previous surgical resection or irradiation. This study analyzed 102 patients, comprising 78 with glioblastomas, *IDH*-wildtype, CNS WHO grade 4; and 24 with *IDH*-mutant gliomas (9 with astrocytoma, *IDH*-mutant, CNS WHO grade 2; 3 with astrocytoma, *IDH*-mutant, CNS WHO grade 3; 1 with astrocytoma, *IDH*-mutant, CNS WHO grade 4; 6 with oligodendroglioma, *IDH*-mutant and *1p/19q-*codeleted, CNS WHO grade 2; and 5 with oligodendroglioma, *IDH*-mutant and *1p/19q-*codeleted, CNS WHO grade 3) (Fig. 1). All glioblastomas, *IDH*-wildtype, CNS WHO grade 4, were enhancing lesions, except for one case. One case of astrocytoma, *IDH*-mutant, CNS WHO grade 4, was an enhancing lesion, whereas all astrocytomas, *IDH*-mutant, CNS WHO grades 3 and 2, were nonenhancing lesions. Three and two of oligodendrogliomas, *IDH*-mutant and *1p/19q-*codeleted, CNS WHO grade 3, were enhancing and nonenhancing lesions, respectively. All oligodendrogliomas, *IDH*-mutant and *1p/19q*-codeleted, CNS WHO grade 2, were nonenhancing lesions. All patients were pathologically diagnosed after total or partial surgical resection. Table 1 presents the patients’ demographic and pathological characteristics. A previous study compared glioblastomas and primary central nervous system lymphomas using time-dependent diffusion MRI [26]. Patients with glioblastoma analyzed in the present study involved 66 individuals who were included in our previous study. Sex did not differ among the three groups (*p* = 0.692). Age, CNS WHO grade, Ki-67 LI, and time interval between MRI and surgery significantly differed among the three groups (each *p* < 0.001). Figures 2 and 3 illustrate the representative diffusion parametric maps of *IDH*-mutant glioma (LGG) and *IDH*-wildtype glioma (HGG).

**Table 1:** Patient demographic and pathological characteristics of patients

Characteristics	IDH-mutant	IDH-mutant	IDH-wildtype	*p* value
	1p/19q-codeleted (*n* = 11)	1p/19q-intact (*n* = 13)	(*n* = 78)	
Age (years)	50 ± 10	42 ± 14	70 ± 13	< 0.001
Sex (male/female)	7/4	9/4	45/33	0.692
CNS WHO grade Enhancement status				< 0.001
Grade 2 enhanced/nonenhanced	6 (5.9%) 0/6 (0%/5.9%)	9 (8.8%) 0/9 (0%/8.8%)	0	
Grade 3 enhanced/nonenhanced	5 (4.9%) 3/2 (2.9%/2.0%)	3 (2.9%) 0/3 (0%/2.9%)	0	
Grade 4 enhanced/nonenhanced	0	1 (1.0%) 1/0 (1.0%/0%)	78 (76.5%) 77/1 (75.5%/1.0%)	
Ki-67 LI (%)	12.73 ± 11.50	9.53 ± 7.09	39.87 ± 19.10	< 0.001
Time interval between MRI and surgery (days)	9.45 ± 6.89	34.77 ± 56.24	6.03 ± 5.86	< 0.001

Values are expressed as numbers or mean ± standard deviation

*1p/19q* chromosome arms 1p and 19q, *IDH* isocitrate dehydrogenase, *Ki-67* LI Ki-67 labeling index

### Interobserver agreement

Supplementary Table 2 presents the ICCs and 95% confidence intervals (CIs) for each parameter. All parameters demonstrated excellent agreement.

### Assessment of the diffusion parameters between LGGs and HGGs and among tumor grades

**Fig. 4:**
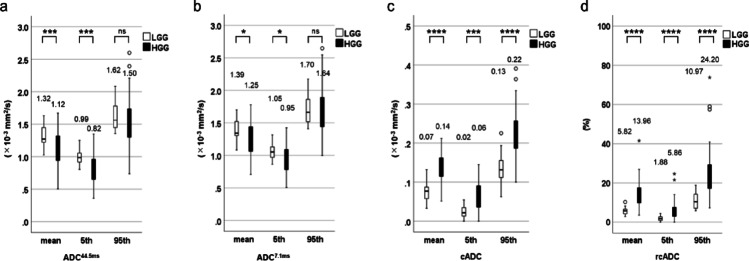
Box and whisker plots of the mean, 5th percentile, and 95th percentile of the ADC_44.5ms_ (**a**), ADC_7.1ms_ (**b**), cADC (**c**), and rcADC (**d**) between pulsed-gradient spin-echo DWI and oscillating-gradient spin-echo DWI for LGGs and HGGs. The mean and 5th percentile of ADC_44.5ms_ and ADC_7.1ms_ were significantly lower for HGGs than for LGGs, and no significant difference was observed between LGGs and HGGs in the 95th percentile of ADC_44.5ms_ and ADC_7.1ms_ (**a**,** b**). All three indices of the cADC and rcADC values were significantly higher for HGGs than for LGGs (**c**, **d**). *p < 0.05, **p < 0.01, ***p < 0.005, ****p < 0.001 *ADC* apparent diffusion coefficient, *cADC* apparent diffusion coefficient change, *DWI* diffusion-weighted imaging, *HGG* high-grade gliomas, *LGG* low-grade gliomas, *rcADC* relative apparent diffusion coefficient change

**Fig. 5:**
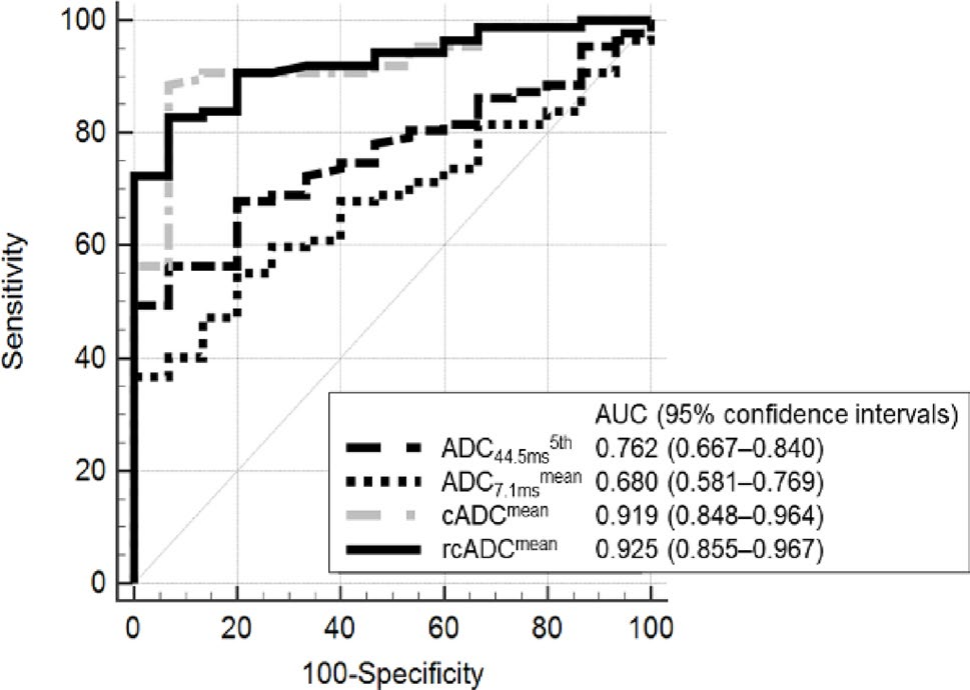
Receiver operating characteristic curves for the best-performing ADC_44.5ms_, ADC_7.1ms_, cADC, and rcADC indices in the solid tumor component to differentiate between low-grade and high-grade gliomas *cADC* apparent diffusion coefficient change, *rcADC* relative apparent diffusion coefficient change

The mean and 5th percentile of the ADC_44.5ms_ and ADC_7.1ms_ were significantly lower for HGGs than for LGGs (*p* = 0.003 for the ADC_44.5ms_^mean^, *p* = 0.001 for the ADC_44.5ms_^5th^, *p* = 0.026 for the cADC^mean^, and *p* = 0.029 for the cADC^5th^), with no significant difference between the LGGs and HGGs in the 95th percentile of the ADC_44.5ms_ and ADC_7.1ms_ (*p* = 0.120 and 0.422) (Fig. 4a and b). All three indices for the cADC and rcADC values were significantly higher for the HGGs than for the LGGs (each *p* < 0.001) (Fig. 4c and d). Table 2 presents the results of the AUC, optimal threshold, sensitivity, specificity, and accuracy for the diffusion parameters of solid tumor components for differentiating LGGs and HGGs. The ADC_44.5ms_^5th^ (AUC: 0.762; 95% CI: 0.667–0.840), ADC_7.1ms_^mean^ (0.680; 0.581–0.769), cADC^mean^ (0.919; 0.848–0.964), and rcADC^mean^ (0.925; 0.855–0.967) values were the best-performing indices for ADC_44.5ms_, ADC_7.1ms_, cADC, and rcADC, respectively. Figure 5 illustrates the ROC curves for ADC_44.5ms_^5th^, ADC_7.1ms_^mean^, cADC^mean^, and rcADC^mean^ values. The pairwise comparisons of AUCs among the best-performing indices revealed significantly higher AUCs of rcADC^mean^ and cADC^mean^ than those of ADC_44.5ms_^5th^ (AUC, 0.925, 0.919, and 0.762; *p* = 0.0015, 0.0104) (Tables 2 and 3). Age, sex, ADC_44.5ms_^5th^, and rcADC^mean^ were included in the model for the multivariable analysis predicting HGGs. In the multivariable model for predicting HGGs, age and the rcADC^mean^ were retained as independent predictors. The adjusted odds ratio (OR) for age was 1.085 (95% CI: 1.027–1.146, *p* = 0.003), and that for the rcADC^mean^ was 1.652 (95% CI: 1.194–2.285, *p* = 0.002). The model achieved an AUC of 0.959 (95% CI: 0.923–0.996) with a classification accuracy of 91.2%. The stability of the coefficients was confirmed using bootstrap resampling (B = 1,000). Good calibration was confirmed using the Hosmer–Lemeshow test (*p* = 0.941).

**Table 2:** The AUC, optimal threshold, sensitivity, specificity, and accuracy for the ADC_44.5ms_^mean^, ADC_44.5 ms_^5th^, ADC_44.5 ms_^95th^, ADC_7.1 ms_^mean^, ADC_7.1ms_^5th^, ADC_7.1ms_^95th^, cADC^mean^, cADC^5th^, cADC^95th^, rcADC^mean^, rcADC^5th^, and rcADC^95th^ of the solid tumor component to differentiate between the low-grade and high-grade gliomas

Parameters	LGG (*n* = 15)	HGG (*n* = 87)	*p*-value	AUC (95% CI)	Threshold value	Sensitivity (%)	Specificity (%)	Accuracy (%)
ADC_44.5 ms_^mean^	1.32 ± 0.18 (×10^− 3^ mm^2^/s)	1.12 ± 0.25 (×10^− 3^ mm^2^/s)	0.003	0.743 (0.646–0.824)	1.109 (×10^− 3^ mm^2^/s)	52.9	93.3	58.8
ADC_44.5 ms_^5th^	0.99 ± 0.12 (×10^− 3^ mm^2^/s)	0.82 ± 0.21 (×10^− 3^ mm^2^/s)	0.001	0.762 (0.667–0.840)	0.862 (×10^− 3^ mm^2^/s)	56.3	93.3	61.8
ADC_44.5 ms_^95th^	1.62 ± 0.23 (×10^− 3^ mm^2^/s)	1.50 ± 0.30 (×10^− 3^ mm^2^/s)	0.120	0.626 (0.525–0.720)	1.35 (×10^− 3^ mm^2^/s)	34.5	100	44.1
ADC_7.1 ms_^mean^	1.39 ± 0.18 (×10^− 3^ mm^2^/s)	1.25 ± 0.24 (×10^− 3^ mm^2^/s)	0.026	0.680 (0.581–0.769)	1.289 (×10^− 3^ mm^2^/s)	57.5	80.0	60.8
ADC_7.1 ms_^5th^	1.05 ± 0.12 (×10^− 3^ mm^2^/s)	0.95 ± 0.20 (×10^− 3^ mm^2^/s)	0.029	0.677 (0.578–0.767)	0.862 (×10^− 3^ mm^2^/s)	37.9	100	47.1
ADC_7.1 ms_^95th^	1.70 ± 0.24 (×10^− 3^ mm^2^/s)	1.64 ± 0.29 (×10^− 3^ mm^2^/s)	0.422	0.565 (0.463–0.663)	1.433 (×10^− 3^ mm^2^/s)	253	93.3	35.3
cADC^mean^	0.07 ± 0.02 (×10^− 3^ mm^2^/s)	0.14 ± 0.04 (×10^− 3^ mm^2^/s)	< 0.001	0.919 (0.848–0.964)	0.093 (×10^− 3^ mm^2^/s)	88.5	93.3	89.2
cADC^5th^	0.02 ± 0.02 (×10^− 3^ mm^2^/s)	0.06 ± 0.04 (×10^− 3^ mm^2^/s)	< 0.001	0.810 (0.721–0.881)	0.055 (×10^− 3^ mm^2^/s)	60.9	100	66.7
cADC^95th^	0.13 ± 0.05 (×10^− 3^ mm^2^/s)	0.22 ± 0.06 (×10^− 3^ mm^2^/s)	< 0.001	0.882 (0.803–0.937)	0.157 (×10^− 3^ mm^2^/s)	87.4	80.0	86.3
rcADC^mean^	5.82 ± 2.01 (%)	13.96 ± 5.94 (%)	< 0.001	0.925 (0.855–0.967)	8.25 (%)	82.8	93.3	84.3
rcADC^5th^	1.88 ± 1.32 (%)	5.86 ± 4.38 (%)	< 0.001	0.816 (0.727–0.886)	4.45 (%)	60.9	100	66.7
rcADC^95th^	10.97 ± 4.46 (%)	24.20 ± 10.95 (%)	< 0.001	0.905 (0.831–0.954)	17.35 (%)	74.7	93.3	77.5

Values are expressed as mean ± standard deviation

*ADC* apparent diffusion coefficient, *AUC* area under the receiver operating characteristic curve, *cADC* apparent diffusion coefficient change, *HGG* high-grade glioma, *LGG* low-grade glioma, *rcADC* relative apparent diffusion coefficient change

**Table 3:** Pairwise comparison of the AUCs among the ADC_44.5ms_^5th^, ADC_7.1ms_^mean^, cADC^mean^, and rcADC^mean^ in the solid tumor component to differentiate between low-grade and high-grade gliomas

Parameter	rcADC^mean^	cADC^mean^	ADC_7.1ms_^mean^
ADC_44.5 ms_^5th^ DBA p	0.163 0.0015	0.157 0.0104	0.081 0.0955
ADC_7.1 ms_^mean^ DBA p	0.244 0.0001	0.239 0.0012	
cADC^mean^ DBA p	0.005 0.7658		

*AUC* area under the receiver operating characteristic curve, *cADC* apparent diffusion coefficient change, *DBA* difference between area under the receiver operating characteristic curve for each pair, *rcADC* relative apparent diffusion coefficient change

Supplementary Tables 3 and visualized in Supplementary Fig. 2 present the comparisons of diffusion parameters according to tumor grade. Significant differences in all ADC_44.5 ms_ indices among the three tumor grades were observed (*p* = 0.002 for the ADC_44.5 ms_^mean^, *p* = 0.003 for the ADC_44.5ms_^5th^, and *p* = 0.037 for the ADC_44.5 ms_^95th^). The ADC_7.1ms_^mean^ demonstrated significant differences among the three tumor grades (*p* = 0.011), except for the ADC_7.1 ms_^5th^ and ADC_7.1 ms_^95th^ (*p* = 0.052 and 0.054). The ADC_44.5ms_^mean^, ADC_44.5 ms_^5th^, and ADC_7.1 ms_^mean^ values were significantly lower for CNS WHO grade 4 than for CNS WHO grade 2 (*p* = 0.004, 0.005, and 0.048, respectively). All three indices of the cADC and rcADC were significantly higher for CNS WHO grade 4 than for CNS WHO grade 2 (*p* = 0.001 for the cADC^5th^, *p* = 0.004 for the rcADC^5th^, and *p* < 0.001 for the others, respectively). No significant differences in any diffusion parameters were found between CNS WHO grades 3 and 4. Moreover, no significant difference in all the ADC_44.5ms_ and ADC_7.1 ms_ indices was observed between CNS WHO grades 2 and 3. However, cADC^mean^, cADC^5th^, cADC^95th^, rcADC^mean^, and rcADC^5th^ were significantly higher CNS WHO grade 3 than for grade 2 (*p* < 0.001, 0.002, 0.004, 0.018, and 0.015, respectively), except for the cADC^95th^ (*p* = 0.175).

### Assessment of diffusion parameters between *IDH*-mutant and *IDH*-wildtype gliomas and among tumor subtypes

**Fig. 6:**
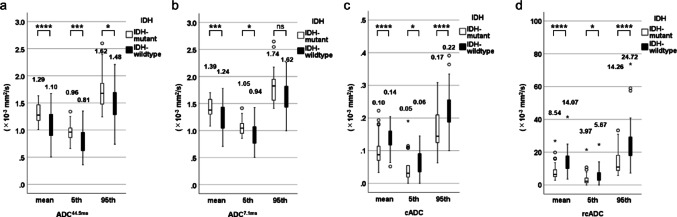
Box and whisker plots of the mean, 5th percentile, and 95th percentile of ADC_44.5ms_ (**a**), ADC_7.1ms_ (**b**), cADC (**c**), and rcADC (**d**) between pulsed-gradient spin-echo and oscillating gradient spin-echo diffusion-weighted imaging for *isocitrate dehydrogenase* (*IDH*)-mutant and *IDH*-wildtype gliomas. *p < 0.05, **p < 0.01, ***p < 0.005, ****p < 0.001 *cADC* apparent diffusion coefficient change, *rcADC* relative apparent diffusion coefficient change

**Fig. 7:**
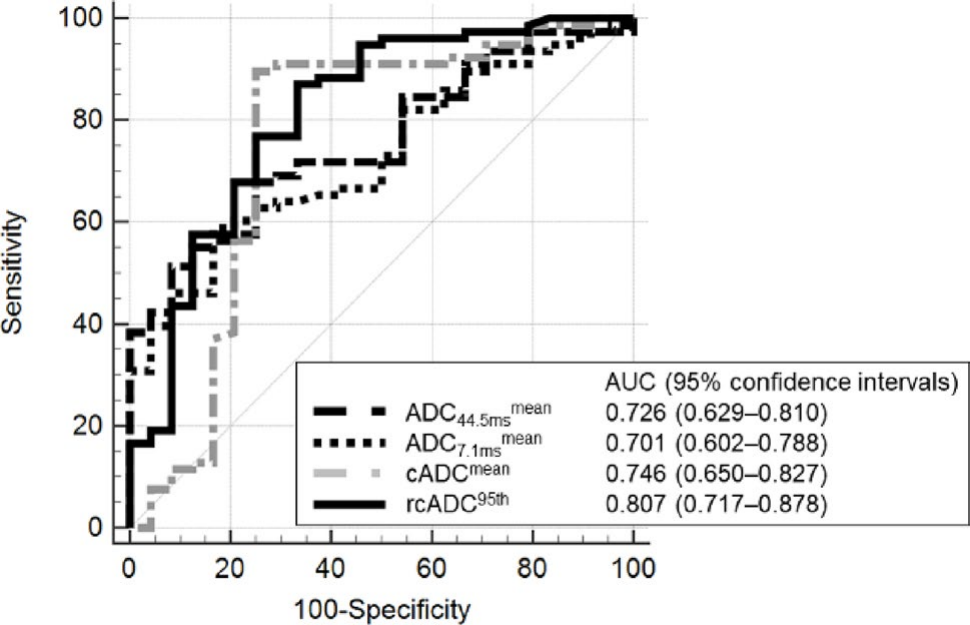
Receiver operating characteristic curves for the best-performing ADC_44.5ms_, ADC_7.1ms_, cADC, and rcADC indices in the solid tumor component to differentiate between *isocitrate dehydrogenase*-mutant and *isocitrate dehydrogenase*-wildtype gliomas *cADC* apparent diffusion coefficient change, *rcADC* relative apparent diffusion coefficient change

All indices for ADC_44.5ms_ and ADC_7.1ms_ were significantly lower for *IDH*-wildtype gliomas than for *IDH*-mutant gliomas (*p* < 0.001 for the ADC_44.5 ms_^mean^, *p* = 0.001 for the ADC_44.5ms_^5th^, *p* = 0.029 for the ADC_44.5 ms_^95th^, *p* = 0.003 for the ADC_7.1ms_^mean^, and *p* = 0.012 for the ADC_7.1ms_^5th^), except for ADC_7.1 ms_^95th^ (*p* = 0.067) (Figs. 6a and b). All three indices for the cADC and rcADC values were significantly higher for *IDH*-wildtype gliomas than for *IDH*-mutant gliomas (*p* < 0.001 for cADC^mean^, cADC^95th^, rcADC^mean^, and rcADC^95th^; *p* = 0.032 for the cADC^5th^; and *p* = 0.015 for the rcADC^5th^) (Fig. 6c and d). Table 4 presents the AUC, optimal threshold, sensitivity, specificity, and accuracy for diffusion parameters of solid tumor components to differentiate between *IDH*-mutant and *IDH*-wildtype gliomas. The ADC_44.5ms_^mean^, ADC_7.1 ms_^mean^, cADC^mean^, and rcADC^95th^ values were the best-performing indices for ADC_44.5ms_, ADC_7.1 ms_, cADC, and rcADC, respectively. Figure 7 illustrates the ROC curves for the ADC_44.5 ms_^mean^ (AUC: 0.726, 95% CI: 0.629–0.810), ADC_7.1ms_^mean^ (0.701, 0.602–0.788), cADC^mean^ (0.746, 0.650–0.827), and rcADC^95th^ (0.807, 0.717–0.878) values. Pairwise comparisons of AUCs among the best-performing indices revealed that despite the highest performance of rcADC^95th^ (AUC: 0.807, 95% CI: 0.717–0.878), none of the AUC comparisons revealed significant differences (Table 5). At first, age, sex, ADC_44.5ms_^mean^, and rcADC^95th^ were included in the model for the multivariable analysis predicting *IDH*-wildtype gliomas. In the multivariable model for predicting *IDH*-wildtype gliomas, age and the rcADC^95th^ were retained as independent predictors. The adjusted OR for age was 1.125 (95% CI: 1.065–1.190, *p* < 0.001), and that for the rcADC^95th^ was 1.149 (95% CI: 1.026–1.286, *p* = 0.016). The model achieved an AUC of 0.927 (95% CI: 0.872–0.983) with a classification accuracy of 92.2%. The stability of the coefficients was confirmed using bootstrap resampling (B = 1,000). Good calibration was confirmed by the Hosmer–Lemeshow test (*p* = 0.681). Second, CNS WHO grade was added to the variables included in the multivariable analysis, in addition to age, sex, ADC_44.5ms_^mean^, and rcADC^95th^. None of the variables, including age and rcADC^95th^, were independent predictors. The adjusted OR for age was 7.787 (95% CI: 0–7.068E + 101, *p* = 0.981), and that for the rcADC^95th^ was 52.214 (95% CI: 0–1.884E + 216, *p* = 0.987).

**Table 4:** The AUC, optimal threshold, sensitivity, specificity, and accuracy for the ADC_44.5 ms_^mean^, ADC_44.5ms_^5th^, ADC_44.5 ms_^95th^, ADC_7.1 ms_^mean^, ADC_7.1 ms_^5th^, ADC_7.1 ms_^95th^, cADC^mean^, cADC^5th^, cADC^95th^, rcADC^mean^, rcADC^5th^, and rcADC^95th^ of the solid tumor component to differentiate between the IDH-mutant and IDH-wildtype gliomas

Parameters	IDH-mutant (*n* = 24)	IDH-wildtype (*n* = 78)	*p*	AUC (95% CI)	Threshold value	Sensitivity (%)	Specificity (%)	Accuracy (%)
ADC_44.5 ms_^mean^	1.29 ± 0.20 (×10^− 3^ mm^2^/s)	1.10 ± 0.24 (×10^− 3^ mm^2^/s)	< 0.001	0.726 (0.629–0.810)	1.181 (×10^− 3^ mm^2^/s)	65.4	75.0	67.6
ADC_44.5 ms_^5th^	0.96 ± 0.16 (×10^− 3^ mm^2^/s)	0.81 ± 0.21 (×10^− 3^ mm^2^/s)	0.001	0.718 (0.621–0.803)	0.795 (×10^− 3^ mm^2^/s)	50.0	91.7	59.8
ADC_44.5 ms_^95th^	1.62 ± 0.26 (×10^− 3^ mm^2^/s)	1.48 ± 0.30 (×10^− 3^ mm^2^/s)	0.029	0.647 (0.547–0.739)	1.41 (×10^− 3^ mm^2^/s)	47.4	83.3	55.9
ADC_7.1 ms_^mean^	1.39 ± 0.18 (×10^− 3^ mm^2^/s)	1.24 ± 0.23 (×10^− 3^ mm^2^/s)	0.003	0.701 (0.602–0.788)	1.277 (×10^− 3^ mm^2^/s)	59.0	79.2	63.7
ADC_7.1 ms_^5th^	1.05 ± 0.15 (×10^− 3^ mm^2^/s)	0.94 ± 0.20 (×10^− 3^ mm^2^/s)	0.012	0.671 (0.571–0.761)	0.852 (×10^− 3^ mm^2^/s)	38.5	100	52.9
ADC_7.1 ms_^95th^	1.74 ± 0.24 (×10^− 3^ mm^2^/s)	1.62 ± 0.29 (×10^− 3^ mm^2^/s)	0.067	0.624 (0.523–0.718)	1.653 (×10^− 3^ mm^2^/s)	60.3	66.7	61.8
cADC^mean^	0.10 ± 0.05 (×10^− 3^ mm^2^/s)	0.14 ± 0.04 (×10^− 3^ mm^2^/s)	< 0.001	0.746 (0.650–0.827)	0.095 (×10^− 3^ mm^2^/s)	89.7	75.0	86.3
cADC^5th^	0.05 ± 0.05 (×10^− 3^ mm^2^/s)	0.06 ± 0.04 (×10^− 3^ mm^2^/s)	0.032	0.646 (0.545–0.738)	0.055 (×10^− 3^ mm^2^/s)	61.5	79.2	65.7
cADC^95th^	0.17 ± 0.07 (×10^− 3^ mm^2^/s)	0.22 ± 0.06 (×10^− 3^ mm^2^/s)	< 0.001	0.741 (0.644–0.822)	0.163 (×10^− 3^ mm^2^/s)	85.9	62.5	80.4
rcADC^mean^	8.54 ± 5.88 (%)	14.07 ± 5.79 (%)	< 0.001	0.796 (0.704–0.869)	8.25 (%)	85.9	75.0	83.3
rcADC^5th^	3.97 ± 4.72 (%)	5.67 ± 4.12 (%)	0.015	0.665 (0.565–0.755)	4.45 (%)	61.5	79.2	65.7
rcADC^95th^	14.26 ± 7.84 (%)	24.72 ± 11.05 (%)	< 0.001	0.807 (0.717–0.878)	14.75 (%)	87.2	66.7	82.4

Values are expressed as mean ± standard deviation

*AUC* area under the receiver operating characteristic curve, *cADC* apparent diffusion coefficient change, *IDH* isocitrate dehydrogenase, *rcADC* relative apparent diffusion coefficient change

**Table 5:** Pairwise comparison of AUCs among the ADC_44.5ms_^mean^, ADC_7.1ms_^mean^, cADC^mean^, and rcADC^95th^ in the solid tumor component to differentiate between the IDH-mutant and IDH-wildtype gliomas

Parameter	rcADC^95th^	cADC^mean^	ADC_7.1ms_^mean^
ADC_44.5ms_^mean^ DBA p	0.080 0.0879	0.019 0.7848	0.025 0.1183
ADC_7.1ms_^mean^ DBA p	0.106 0.5901	0.045 0.5901	
cADC^mean^ DBA p	0.061 0.0715		

*ADC* apparent diffusion coefficient, *AUC* area under the receiver operating characteristic curve, *cADC* apparent diffusion coefficient change, *DBA* difference between area under the receiver operating characteristic curve for each pair, *IDH* isocitrate dehydrogenase, *rcADC* relative apparent diffusion coefficient change

Supplementary Tables 4 and Fig. 3 illustrate the comparisons of diffusion parameters among the tumor subtypes. Significant differences in almost all diffusion parameters were observed among the three tumor subtypes (*p* < 0.001 for ADC_44.5ms_^mean^, ADC_44.5ms_^5th^, ADC_7.1ms_^mean^, cADC^mean^, cADC^95th^, rcADC^mean^, and rcADC^95th^, *p* = 0.024 for ADC_44.5ms_^95th^, *p* = 0.008 for ADC_7.1ms_^5th^, *p* = 0.048 for ADC_7.1ms_^95th^, and *p* = 0.032 for rcADC^5th^), except for cADC^5th^ (*p* = 0.067). ADC_44.5ms_^mean^, ADC_44.5ms_^5th^, ADC_44.5ms_^95th^, ADC_7.1ms_^mean^, and ADC_7.1ms_^5th^ were significantly lower for glioblastomas, *IDH*-wildtype, than for astrocytoma, *IDH*-mutant (*p* < 0.001, < 0.001, 0.022, < 0.001, and 0.006, respectively), except for ADC_7.1ms_^95th^ (*p* = 0.056). Moreover, cADC^mean^, cADC^95th^, rcADC^mean^, and rcADC^95th^ were significantly higher for glioblastomas, *IDH*-wildtype, than for astrocytoma, *IDH*-mutant (each *p* < 0.001), except for cADC^5th^ and rcADC^5th^ (*p* = 0.129 and 0.103). ADC_44.5ms_^mean^ and ADC_7.1ms_^mean^ values were significantly lower in the oligodendroglioma, *IDH*-mutant and *1p/19q*-codeleted than in the astrocytoma, *IDH*-mutant (*p* = 0.017 and 0.028, respectively). No significant differences in other parameters were observed between the two tumor types. Furthermore, no significant differences in any diffusion parameters were found between glioblastomas, *IDH*-wildtype, and oligodendroglioma, *IDH*-mutant and *1p/19q*-codeleted.

### Correlation between diffusion parameters and Ki-67 LI

**Fig. 8:**
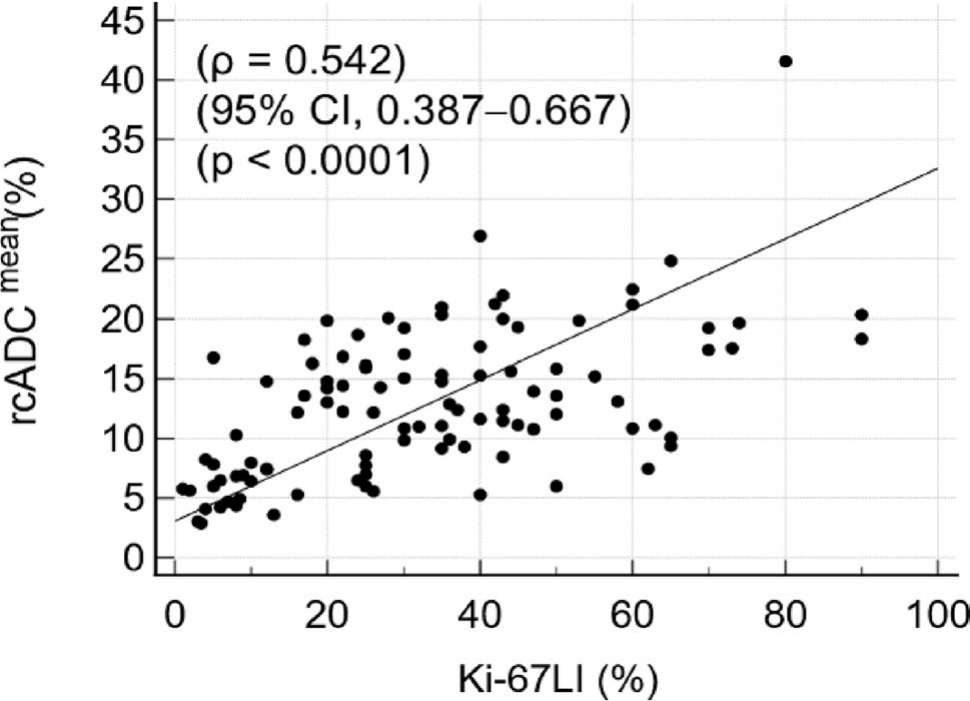
Scatter plot illustrates the association between Ki-67 labeling index and rcADC. Significant positive correlation was noted between these parameters *rcADC* relative apparent diffusion coefficient change

The Ki-67 LI was significantly negatively correlated with ADC_44.5ms_^mean^, ADC_44.5ms_^5th^, ADC_7.1ms_^mean^, and ADC_7.1ms_^5th^, which was considered little to fair, except for ADC_44.5ms_^95th^ and ADC_7.1ms_^95th^. Moreover, it was significantly positively correlated with all cADC and rcADC indices, which were considered fair to good (Supplementary Table 5). The rcADC^mean^ demonstrated the strongest correlation with the Ki-67 LI in these diffusion indices. Figure 8 illustrates the scatter plot of Ki-67 LI and rcADC^mean^.

## Discussion

This study revealed significantly higher time-dependent diffusion MRI parameters, including both the cADC and rcADC, in HGGs than in LGGs. The AUC of rcADC^mean^ for differentiating between LGGs and HGGs was significantly higher than the AUCs of any conventional ADC-based index. Moreover, both cADC and rcADC were significantly higher for *IDH*-wildtype gliomas than for *IDH*-mutant gliomas. Of all indices, rcADC, particularly rcADC^95th^, demonstrated the highest differentiating performance, although its superiority to conventional ADC-based indices was not statistically proven.

The clinical value of ADC in distinguishing HGGs from LGGs has been well documented. A distinctly low ADC in the solid tumor component of HGGs, which indicates higher cellularity and increased nuclear-to-cytoplasmic ratio, helps differentiate HGGs from LGGs [10−13]. Moreover, the ADC values distinguish *IDH*-wildtype gliomas from *IDH*-mutant gliomas. However, regarding the *1p/19q* codeletion status, the value of the ADC measurement remains unidentified [14−16].

Studies have investigated the application of time-dependent diffusion MRI in evaluating intracranial tumors. Maekawa et al. have demonstrated significantly higher cADC and rcADC between the short (6.5 ms) and long (32.5 ms) effective diffusion times in the high-grade intra-axial brain tumors than in the low-grade tumors [24]. More recently, Zhang et al. investigated pediatric gliomas using time-dependent diffusion MRI and used a two-compartment microstructural model to identify the intracellular fraction, cell diameter, and cellularity [33]. They revealed that the cellularity index provided the best performance in identifying the histological grade, whereas the cell diameter offered the most accurate differentiation for the molecular classification of *H3K27*-altered gliomas in midline gliomas. Moreover, Zhu et al. investigated five patients with glioma using an ultra-high-performance gradient MRI system and demonstrated that the ratio of the ADC measured at short diffusion times to that at long diffusion times holds promise for revealing heterogeneous tumor microstructures, including cellular density, in both presurgical and post-treatment gliomas [34]. These studies have revealed the clinical potential and validity of time-dependent diffusion MRI for profiling intracranial tumors. However, no research has fully assessed the diagnostic performance of time-dependent diffusion MRI parameters in distinguishing HGGs from LGGs and *IDH*-wildtype gliomas from *IDH*-mutant gliomas compared with conventional ADC.

ADC_44.5ms_^mean^, ADC_44.5ms_^5th^, ADC_7.1ms_^mean^, and ADC_7.1ms_^5th^ values were significantly lower for HGGs than for LGGs, which is consistent with the results of previous studies [10−13], and all three indices of the cADC and rcADC values were significantly higher for HGGs than for LGGs. The pairwise comparisons of the AUCs for differentiating LGGs from HGGs revealed that the AUC of rcADC^mean^ was significantly higher than that of ADC_44.5ms_^5th^, which was the best-performing ADC-based index. The differentiation of the three tumor grades revealed a significant difference in the conventional ADC_44.5ms_^mean^ between CNS WHO grades 2 and 4, despite no significant difference between CNS WHO grades 2 and 3 and between CNS WHO grades 3 and 4. Conversely, almost all cADC and rcADC parameters demonstrated significant differences between CNS WHO grades 2 and 3, except for rcADC^95th^.

The Ki-67 LI serves as a marker of tumor cell proliferation, with increased values reflecting evaluated proliferative activity [35]. The present study revealed that the Ki-67 LI was negatively correlated with almost all ADC indices, except for ADC_44.5ms_^95th^ and ADC_7.1ms_^95th^, and positively correlated with all cADC and rcADC indices. Moreover, the rcADC^mean^ demonstrated the strongest correlation, indicating that rcADC^mean^ derived from time-dependent diffusion MRI may provide a more accurate assessment of glioma cell proliferation, which may explain the better differentiation performance between LGGs and HGGs.

The differentiation between *IDH*-mutant and *IDH*-wildtype gliomas indicated that all three indices of ADC_44.5ms_ and ADC_7.1ms_ were significantly lower for *IDH*-wildtype gliomas than for *IDH*-mutant gliomas. The ADC_44.5ms_^mean^ and ADC_7.1ms_^mean^ values of oligodendrogliomas (*IDH*-mutant and *1p/19q*-codeleted gliomas) were significantly lower than those of astrocytomas (*IDH*-mutant gliomas with intact *1p/19q*). These results are consistent with those of previous studies [14−16]. All *IDH*-wildtype gliomas are classified as CNS WHO grade 4, which is characterized by a higher cellularity [2]. This may explain why *IDH*-wildtype gliomas have lower ADC values than *IDH*-mutant gliomas. However, why intermediate ADC values were observed in the *1p/19q*-codeleted gliomas remains unknown. Regarding the time-dependent diffusion MRI parameters, all three indices of the cADC and rcADC values were significantly higher for *IDH*-wildtype gliomas than for *IDH*-mutant gliomas. A similar trend was observed across all three indices of cADC and rcADC values in differentiating LGGs from HGGs. However, the diagnostic performance of rcADC was reduced. The *IDH*-mutant gliomas include HGGs such as astrocytoma, *IDH*-mutant, CNS WHO grade 3 and 4, and oligodendroglioma, *IDH*-mutant and *1p/19q-*codeleted, CNS WHO grade 3. The pathological features of high-grade *IDH*-mutant and *IDH*-wildtype gliomas show considerable overlap [2]. These tumors are associated with higher cellularity and an increased nuclear-to-cytoplasmic ratio compared with CNS WHO grade 2 tumors [2], which may have led to higher rcADC values. Age, sex, ADC_44.5ms_^mean^, and rcADC^95th^ were included in the model for the multivariable analysis predicting *IDH*-wildtype gliomas, in which rcADC^95th^ and age were identified as independent predictors. Furthermore, CNS WHO grade was added to the variables included in the multivariable analysis, none of the variables, including age and rcADC^95th^, were independent predictors. This may be due to a strong correlation between CNS WHO grade and imaging markers or age, as well as the influence of multicollinearity resulting from the limited number of cases. From a clinical application perspective, CNS WHO grade is not identified preoperatively; therefore, a predictive model based on imaging markers without including CNS WHO grade would be more practical. In this study, rcADC^95th^, as an independent predictor of *IDH*-wildtype gliomas after adjusting for age and sex was useful for distinguishing *IDH*-wildtype from *IDH*-mutant gliomas.

### Limitations of the study

This study has several limitations. First, the sample size was relatively small. Thus, studies with larger sample sizes are warranted to confirm our findings. Second, we measured ROIs under two different conditions, including enhancing and nonenhancing compartments. This may be affecting the ADC measurement. A more standardized method for setting ROIs is expected. Third, only two effective diffusion times (7.1 ms and 44.5 ms) and a fixed set of b-values (0 and 1,500 s/mm^2^) were examined. The use of shorter or longer effective diffusion times may have affected the results. However, the gradient performance of our clinical MRI system limited the range of effective diffusion time in OGSE. An MRI system with higher gradients may help achieve a shorter effective diffusion time in OGSE. Finally, all tumors were pathologically diagnosed; however, the detailed comparisons between the tissue microstructures and imaging findings were not performed.

## Conclusions

The study demonstrates that rcADC indices derived from time-dependent diffusion MRI outperform conventional PGSE-based ADC parameters in glioma characterization. Specifically, rcADC^mean^ shows a strong correlation with Ki-67 LI and serves as an independent predictor of HGGs, while rcADC^95th^ independently differentiates *IDH*-wildtype from *IDH*-mutant gliomas. These findings highlight the value of rcADC indices for improved noninvasive assessment of glioma grade and molecular status.

## Supplementary Information

Below is the link to the electronic supplementary material.

Supplementary Material 1

Supplementary Material 2
