# Supplementary material for: Time-dependent diffusion-weighted imaging assessment of tumor grading and isocitrate dehydrogenase genotypes in adult-type diffuse gliomas
Source: Jpn J Radiol. 2026 Jan 5;44(5):882–94. doi: 10.1007/s11604-025-01936-w (PMC13144230; doi:10.1007/s11604-025-01936-w)
Supplement: Supplementary file 1 — Supplementary Material 1 [file 11604_2025_1936_MOESM1_ESM.docx]

**Supplementary Table 1**: Imaging parameters of pre- and postcontrast conventional MRI sequences

|  | 2D fluid-attenuated inversion recovery imaging | Pre- and postcontrast  2D T1-weighted imaging |
| --- | --- | --- |
| Sequence | 2D IR-TSE | 2D SE |
| TR (ms) | 9,000 | 520 |
| TE (ms) | 121 | 12 |
| TI (ms) | 2,430 | N/A |
| FA (degree) | 120 | 70/180 |
| Bandwidth (Hz/pixel) | 130 | 181 |
| Number of averages | 1 | 1 |
| Turbo factor | 25 | N/A |
| Acceleration factor | 2 | N/A |
| FOV (mm) | 230 | 230 |
| Matrix | 307 × 384 | 269 × 384 |
| Thickness (mm) | 5 | 5 |
| Intersection gap (mm) | 1 | 1 |
| Acquisition time (s) | 126 | 148 |

*FA* flip angle, *FOV* field of view, *IR-TSE* inversion recovery turbo spin-echo, *SE* spin-echo, *TE* echo time, *TI* inversion time, *TR* repetition time, *N/A* not applicable.

**Supplementary Table 2**: Intraclass correlation coefficients and 95% confidence intervals for the ADC_44.5ms_^mean^, ADC_44.5ms_^5th^, ADC_44.5ms_^95th^, ADC_7.1ms_^mean^, ADC_7.1ms_^5th^, ADC_7.1ms_^95th^, cADC^mean^, cADC^5th^, cADC^95th^, rcADC^mean^, rcADC^5th^, and rcADC^95th^

| Parameters | Intraclass correlation coefficients (95% confidence intervals) |
| --- | --- |
| ADC_44.5ms_^mean^ | 0.974 (0.962–0.982) |
| ADC_44.5ms_^5th^ | 0.909 (0.868–0.937) |
| ADC_44.5ms_^95th^ | 0.967 (0.952–0.978) |
| ADC_7.1ms_^mean^ | 0.972 (0.958–0.981) |
| ADC_7.1ms_^5th^ | 0.904 (0.862–0.934) |
| ADC_7.1ms_^95th^ | 0.960 (0.941–0.973) |
| cADC^mean^ | 0.970 (0.957–0.980) |
| cADC^5th^ | 0.883 (0.832–0.920) |
| cADC^95th^ | 0.910 (0.870–0.938) |
| rcADC^mean^ | 0.974 (0.962–0.982) |
| rcADC^5th^ | 0.930 (0.899–0.952) |
| rcADC^95th^ | 0.954 (0.932–0.968) |

*ADC* apparent diffusion coefficient.

**Supplementary Table 3**: Comparisons of diffusion parameters among the tumor grades of adult-type diffuse gliomas

| Parameters | Grade 2  (n = 15) | Grade 3  (n = 8) | Grade 4  (n = 79) | p  (ANOVA) | p (Grade 2 vs 3) | p (Grade 3 vs 4) | p (Grade 2 vs 4) |
| --- | --- | --- | --- | --- | --- | --- | --- |
| ADC_44.5ms_^mean^ | 1.32 ± 0.18  (×10^−3^ mm^2^/s) | 1.27 ± 0.26  (×10^−3^ mm^2^/s) | 1.10 ± 0.24  (×10^−3^ mm^2^/s) | 0.002 | 1.000 | 0.158 | 0.004 |
| ADC_44.5ms_^5th^ | 0.99 ± 0.12  (×10^−3^ mm^2^/s) | 0.90 ± 0.22  (×10^−3^ mm^2^/s) | 0.81 ± 0.21  (×10^−3^ mm^2^/s) | 0.003 | 0.853 | 0.710 | 0.005 |
| ADC_44.5ms_^95th^ | 1.62 ± 0.23  (×10^−3^ mm^2^/s) | 1.67 ± 0.29  (×10^−3^ mm^2^/s) | 1.48 ± 0.30  (×10^−3^ mm^2^/s) | 0.037 | 1.000 | 0.253 | 0.252 |
| ADC_7.1ms_^mean^ | 1.39 ± 0.18  (×10^−3^ mm^2^/s) | 1.41 ± 0.22  (×10^−3^ mm^2^/s) | 1.24 ± 0.23  (×10^−3^ mm^2^/s) | 0.011 | 1.000 | 0.116 | 0.048 |
| ADC_7.1ms_^5th^ | 1.05 ± 0.12  (×10^−3^ mm^2^/s) | 1.03 ± 0.20  (×10^−3^ mm^2^/s) | 0.94 ± 0.20  (×10^−3^ mm^2^/s) | 0.052 | 1.000 | 0.579 | 0.101 |
| ADC_7.1ms_^95th^ | 1.70 ± 0.24  (×10^−3^ mm^2^/s) | 1.84 ± 0.25  (×10^−3^ mm^2^/s) | 1.62 ± 0.29  (×10^−3^ mm^2^/s) | 0.054 | 0.842 | 0.113 | 0.836 |
| cADC^mean^ | 0.07 ± 0.02  (×10^−3^ mm^2^/s) | 0.14 ± 0.06  (×10^−3^ mm^2^/s) | 0.14 ± 0.04  (×10^−3^ mm^2^/s) | < 0.001 | < 0.001 | 1.000 | < 0.001 |
| cADC^5th^ | 0.02 ± 0.02  (×10^−3^ mm^2^/s) | 0.08 ± 0.06  (×10^−3^ mm^2^/s) | 0.06 ± 0.04  (×10^−3^ mm^2^/s) | < 0.001 | 0.002 | 0.496 | 0.001 |
| cADC^95th^ | 0.13 ± 0.05  (×10^−3^ mm^2^/s) | 0.22 ± 0.07  (×10^−3^ mm^2^/s) | 0.22 ± 0.06  (×10^−3^ mm^2^/s) | < 0.001 | 0.004 | 1.000 | < 0.001 |
| rcADC^mean^ | 5.82 ± 2.01  (%) | 12.68 ± 7.85  (%) | 14.09 ± 5.76  (%) | < 0.001 | 0.018 | 1.000 | < 0.001 |
| rcADC^5th^ | 1.88 ± 1.32  (%) | 7.04 ± 6.56  (%) | 5.74 ± 4.14  (%) | < 0.001 | 0.015 | 1.000 | 0.004 |
| rcADC^95th^ | 10.97 ± 4.46  (%) | 19.56 ± 9.99  (%) | 24.67 ± 10.99  (%) | < 0.001 | 0.175 | 0.544 | < 0.001 |

Values are expressed as mean ± standard deviation.

*ADC* apparent diffusion coefficient, *ANOVA* analysis of variance, *cADC* change in apparent diffusion coefficient, *rcADC* relative change in apparent diffusion coefficient.

**Supplementary Table 4**: Comparison of the diffusion parameters among the adult diffuse glioma tumor subtypes

| Parameters | Oligodendroglioma (subtype 1)  (n = 11) | Astrocytoma (subtype 2)  (n = 13) | Glioblastoma (subtype 3)  (n = 78) | p-value  (ANOVA) | p-value  (subtype 1 vs 2) | p-value  (subtype 2 vs 3) | p-value  (subtype 1 vs 3) |
| --- | --- | --- | --- | --- | --- | --- | --- |
| ADC_44.5ms_^mean^ | 1.15 ± 0.15  (×10^−3^ mm^2^/s) | 1.41 ± 0.16  (×10^−3^ mm^2^/s) | 1.10 ± 0.24  (×10^−3^ mm^2^/s) | < 0.001 | 0.017 | < 0.001 | 1.000 |
| ADC_44.5ms_^5th^ | 0.87 ± 0.13  (×10^−3^ mm^2^/s) | 1.03 ± 0.15  (×10^−3^ mm^2^/s) | 0.81 ± 0.21  (×10^−3^ mm^2^/s) | < 0.001 | 0.121 | < 0.001 | 0.998 |
| ADC_44.5ms_^95th^ | 1.51 ± 0.22  (×10^−3^ mm^2^/s) | 1.72 ± 0.25  (×10^−3^ mm^2^/s) | 1.48 ± 0.30  (×10^−3^ mm^2^/s) | 0.024 | 0.232 | 0.022 | 1.000 |
| ADC_7.1ms_^mean^ | 1.27 ± 0.13  (×10^−3^ mm^2^/s) | 1.50 ± 0.15  (×10^−3^ mm^2^/s) | 1.24 ± 0.23  (×10^−3^ mm^2^/s) | < 0.001 | 0.028 | < 0.001 | 1.000 |
| ADC_7.1ms_^5th^ | 0.97 ± 0.09  (×10^−3^ mm^2^/s) | 1.11 ± 0.16  (×10^−3^ mm^2^/s) | 0.94 ± 0.20  (×10^−3^ mm^2^/s) | 0.008 | 0.189 | 0.006 | 1.000 |
| ADC_7.1ms_^95th^ | 1.64 ± 0.23  (×10^−3^ mm^2^/s) | 1.82 ± 0.24  (×10^−3^ mm^2^/s) | 1.62 ± 0.29  (×10^−3^ mm^2^/s) | 0.048 | 0.345 | 0.056 | 1.000 |
| cADC^mean^ | 0.11 ± 0.06  (×10^−3^ mm^2^/s) | 0.09 ± 0.04  (×10^−3^ mm^2^/s) | 0.14 ± 0.04  (×10^−3^ mm^2^/s) | < 0.001 | 0.430 | < 0.001 | 0.184 |
| cADC^5th^ | 0.06 ± 0.06  (×10^−3^ mm^2^/s) | 0.04 ± 0.04  (×10^−3^ mm^2^/s) | 0.06 ± 0.04  (×10^−3^ mm^2^/s) | 0.067 | 0.674 | 0.129 | 1.000 |
| cADC^95th^ | 0.19 ± 0.07  (×10^−3^ mm^2^/s) | 0.15 ± 0.06  (×10^−3^ mm^2^/s) | 0.22 ± 0.06  (×10^−3^ mm^2^/s) | < 0.001 | 0.394 | < 0.001 | 0.139 |
| rcADC^mean^ | 10.69 ± 7.22  (%) | 6.72 ± 3.88  (%) | 14.07 ± 5.79  (%) | < 0.001 | 0.288 | < 0.001 | 0.214 |
| rcADC^5th^ | 5.19 ± 6.05  (%) | 2.94 ± 3.10  (%) | 5.67 ± 4.12  (%) | 0.032 | 0.597 | 0.103 | 1.000 |
| rcADC^95th^ | 17.39 ± 8.81  (%) | 11.62 ± 6.04  (%) | 24.72 ± 11.05  (%) | < 0.001 | 0.531 | < 0.001 | 0.091 |

Values are expressed as mean ± standard deviation.

*ADC* apparent diffusion coefficient, *AUC* area under the receiver operating characteristic curve, *cADC* apparent diffusion coefficient change, *IDH* isocitrate dehydrogenase, *rcADC* relative apparent diffusion coefficient change, *ANOVA* analysis of variance.

**Supplementary Table 5**: Spearman’s rank correlation coefficient between the diffusion parameters and the Ki-67 labeling index

| Parameters | ρ | 95% confidence interval | p-value |
| --- | --- | --- | --- |
| ADC_44.5ms_^mean^ | −0.311 | −0.477 to −0.123 | 0.0016 |
| ADC_44.5ms_^5th^ | −0.393 | −0.547 to −0.214 | < 0.0001 |
| ADC_44.5ms_^95th^ | −0.133 | −0.320 to −0.064 | 0.1849 |
| ADC_7.1ms_^mean^ | −0.250 | −0.425 to −0.057 | 0.0117 |
| ADC_7.1ms_^5th^ | −0.273 | −0.444 to −0.082 | 0.0058 |
| ADC_7.1ms_^95th^ | −0.069 | −0.261 to 0.128 | 0.4945 |
| cADC^mean^ | 0.480 | 0.314 to 0.618 | < 0.0001 |
| cADC^5th^ | 0.363 | 0.181 to 0.522 | 0.0002 |
| cADC^95th^ | 0.403 | 0.226 to 0.555 | < 0.0001 |
| rcADC^mean^ | 0.542 | 0.387 to 0.667 | < 0.0001 |
| rcADC^5th^ | 0.352 | 0.168 to 0.512 | 0.0003 |
| rcADC^95th^ | 0.520 | 0.362 to 0.650 | < 0.0001 |

*ADC* apparent diffusion coefficient, *cADC* change in apparent diffusion coefficient, *rcADC* relative apparent diffusion coefficient change.
