# Supplementary material for: Time-dependent diffusion-weighted imaging assessment of tumor grading and isocitrate dehydrogenase genotypes in adult-type diffuse gliomas
Source: Jpn J Radiol. 2026 Jan 5;44(5):882–94. doi: 10.1007/s11604-025-01936-w (PMC13144230; doi:10.1007/s11604-025-01936-w)
Supplement: Supplementary file 2 — Supplementary Material 2 [file 11604_2025_1936_MOESM2_ESM.docx]

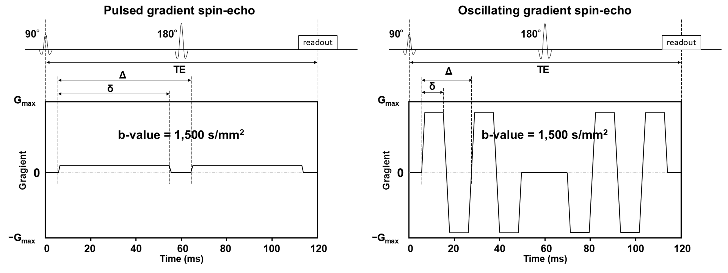


**Fig. 1** Schematic representation of diffusion gradient waveforms for PGSE (left) and OGSE (right). δ, diffusion gradient pulse duration; Δ, diffusion gradient separation; EPI, echo planar imaging; G, gradient vector; OGSE, oscillating gradient spin-echo; PGSE, pulsed-gradient spin-echo; TE, echo time.


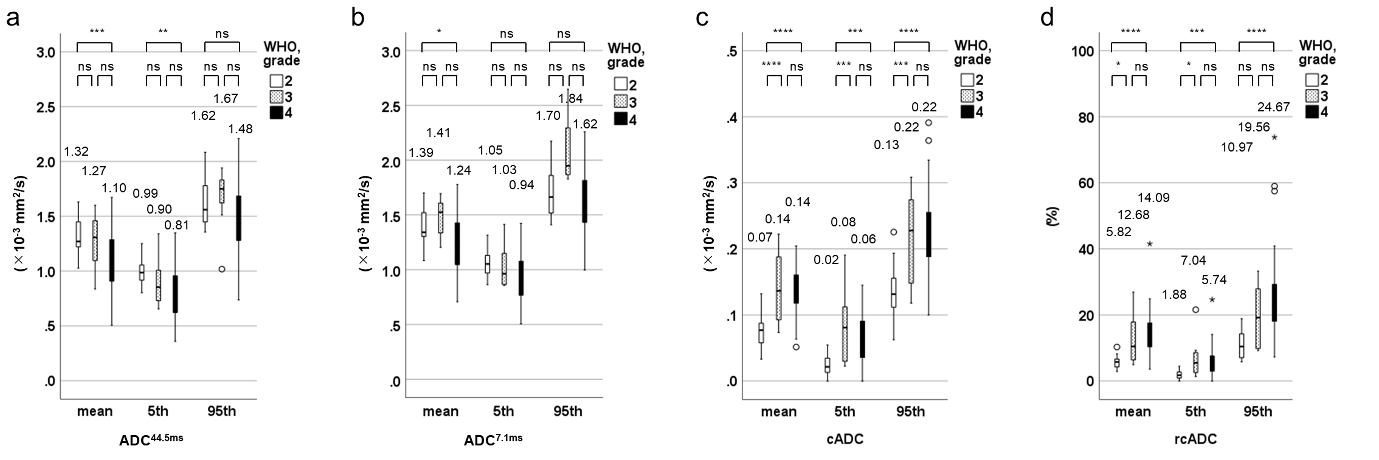


**Fig. 2** Box and whisker plots of the mean, 5th percentile, and 95th percentile of ADC_44.5ms_ (a), ADC_7.1ms_ (b), cADC (c), and rcADC (d) between pulsed-gradient spin-echo and oscillating gradient spin-echo diffusion-weighted imaging for CNS WHO grades 2, 3, and 4. *p < 0.05, **p < 0.01, ***p < 0.005, ****p < 0.001.


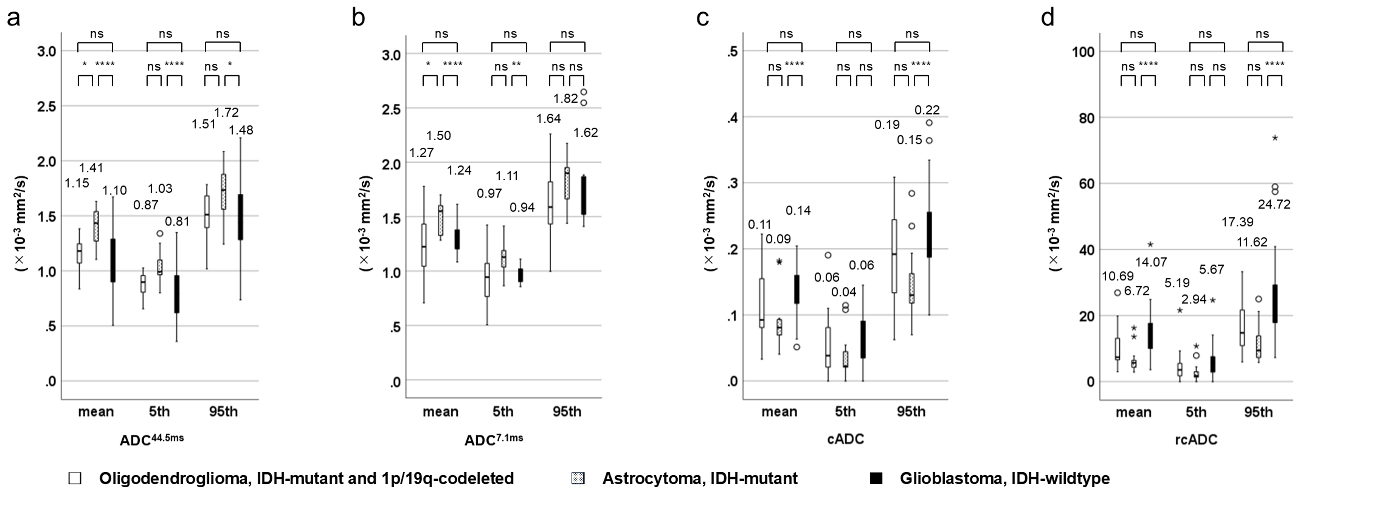


**Fig. 3** Box and whisker plots of the mean, 5th percentile, and 95th percentile of ADC_44.5ms_ (a), ADC_7.1ms_ (b), cADC (c), and rcADC (d) between pulsed-gradient spin-echo and oscillating gradient spin-echo diffusion-weighted imaging for tumor subtypes. *p < 0.05, **p < 0.01, ***p < 0.005, ****p < 0.001.
